# Abundance of Nef and p-Tau217 in Brains of Individuals Diagnosed with HIV-Associated Neurocognitive Disorders Correlate with Disease Severance

**DOI:** 10.1007/s12035-021-02608-2

**Published:** 2021-11-29

**Authors:** Tatiana Pushkarsky, Adam Ward, Andrey Ivanov, Xionghao Lin, Dmitri Sviridov, Sergei Nekhai, Michael I. Bukrinsky

**Affiliations:** 1 The George Washington University School of Medicine and Health Sciences, Washington, DC USA; 2 The George Washington University Milken Institute School of Public Health, Washington, DC USA; 3 Division of Infectious Diseases, Weill Cornell Medicine, New York, NY USA; 4 College of Medicine, Howard University, Washington, DC USA; 5 College of Dentistry, Howard University, Washington, DC USA; 6 Baker Heart and Diabetes Institute, Melbourne, Victoria Australia; 7 Department of Biochemistry and Molecular Biology, Monash University, Clayton, Victoria Australia

**Keywords:** HIV-1, HAND, Nef, p-Tau217, Lipid rafts, ABCA1, Cholesterol

## Abstract

HIV-associated neurocognitive disorders (HAND) is a term used to describe a variety of neurological impairments observed in HIV-infected individuals. The pathogenic mechanisms of HAND and of its connection to HIV infection remain unknown, but one of the considered hypotheses suggests that HIV infection accelerates the development of Alzheimer’s disease. Previous studies suggested that HIV-1 Nef may contribute to HAND by inhibiting cholesterol efflux, increasing the abundance of lipid rafts, and affecting their functionality. Our comparative analysis of postmortem brain samples demonstrated a trend toward the decreased abundance of cholesterol transporter ABCA1 in samples from HIV-infected ART-treated individuals relative to samples from uninfected controls, and a reverse correlation between ABCA1 and flotillin 1, a marker for lipid rafts, in all analyzed samples. The brain samples from HIV-infected individuals, both with and without HAND, were characterized by the increased abundance of p-Tau217 peptide, which correlated with the abundance of flotillin 1. HIV-1 Nef was analyzed in samples from HAND-affected individuals by Western blot with 4 different antibodies and by LC–MS/MS, producing a Nef-positivity score. A significant correlation was found between this score and the abundance of flotillin 1, the abundance of p-Tau217, and the severity of HAND. These results highlight the contribution of Nef and Nef-dependent impairment of cholesterol efflux to HAND pathogenesis and support a connection between the pathogenesis of HAND and Alzheimer’s disease.

**Supplementary Information:**

The online version contains supplementary material available at 10.1007/s12035-021-02608-2.

## Introduction

Although somewhat controversial, HIV-associated neurocognitive disorders (HAND) have clinical hallmarks of a neurodegenerative disease with progressive cognitive decline reflected initially by neuronal dendritic simplification and ultimately by neuronal loss [3]. With the introduction of combination antiretroviral therapy (cART), the prevalence of the severe form of HAND, HIV-associated dementia (HAD), has diminished, but that of milder forms, mild neurocognitive disorder (MND) and especially asymptomatic neurocognitive impairment (ANI), has increased [12], suggesting that cART slows progression, but does not prevent initiation of the disease. The reasons for this persistence are not fully understood and several possible explanations have been suggested (reviewed in [5, 15]). One of the mechanisms gaining support in the recent studies links HAND pathogenesis to beta-amyloid, suggesting a connection between HAND and Alzheimer’s disease (reviewed in [16]). HAND has many similarities to Alzheimer’s disease (AD), such as neuroinflammation, similar transcriptional signatures, and increased abundance and changed localization of intracellular beta-amyloid (Aβ), a critical component in AD pathogenesis [16, 23, 26, 39]. While the possibility that HIV infection “speeds up” the underlying AD is an attractive hypothesis, the mechanisms of this connection remain unclear. Our previous studies of the effects of Nef on cellular cholesterol trafficking and lipid rafts (described below) provide a plausible mechanism.

The expression of Nef in HIV-infected cells induces degradation of the key cellular cholesterol transporter ABCA1, causing suppression of cholesterol efflux and increasing abundance of the lipid rafts [9, 33]. Most importantly, the same activity was demonstrated for the Nef-carrying extracellular vesicles [11, 34], which continue to be released into circulation and the brain from HIV-infected cells even after the suppression of HIV replication by cART [23, 37, 38]. Amyloidogenic proteins concentrate in the lipid rafts [29], and most amyloid proteins involved in the pathogenesis of neurodegeneration are raft proteins [10, 24, 46]. High local concentration of amyloidogenic proteins is a prerequisite for the effective nucleation and cascading progression of their misfolding, a key pathogenic element of neurodegeneration. Therefore, upregulation of the lipid rafts by Nef or Nef-containing extracellular vesicles and accumulation of amyloidogenic proteins in the rafts may accelerate the progress of their misfolding and promote its spread through the brain. A similar mechanism has been shown for prions, which also promote lipid raft formation and depend on lipid rafts for the progression of prion misfolding [8].

In this study, we demonstrated associations between the presence of Nef with reduced levels of ABCA1, increased abundance of flotillin 1, disease severity, and increased abundance of p-Tau217—which is a characteristic marker of Alzheimer’s disease [20, 21, 35]. Our findings support the proposed mechanism of HAND whereby Nef-mediated suppression of ABCA1 increases the abundance of lipid rafts, which in turn enables the progression of tauopathy.

## Materials and Methods

### Brain Samples

Fresh-frozen postmortem samples from the hippocampus or mid-temporal gyrus of HIV-infected individuals with or without HAND diagnosis were obtained from the National NeuroAIDS Tissue Consortium (NNTC). Brain samples from uninfected individuals without neurodegenerative disease diagnosis were obtained from the National Institutes of Health NeuroBioBank (NBB). Clinical information associated with the samples was provided to us by the tissue banks and is presented in Table S1. All samples were deidentified, and no contact between the investigators and sample donors took place. Therefore, the GWU Institutional Review Board (IRB) has determined that this study does not meet the definition of human subject research and does not require review by the GWU IRB. We have abided by all requirements of using the human samples imposed by the NNTC and NBB.

### Western Blot Analysis

Samples were analyzed by automated Western immunoblotting using the Jess™ Simple Western system (ProteinSimple, San Jose, CA). For the analysis of ABCA1, a 66–440-kDa Jess separation module (SM-W008) was used; for Nef, a 2–40-kDa module (SM-W012); and for p-Tau217 and flotillin 1, a 12–230-kDa module (SM-W004). Brain pieces (60 mg per sample) were homogenized in the Triton X-100 RIPA lysis buffer (250 μL, Thermo Fisher) and cleared by centrifugation at 5000 × g. Three microliters of brain lysates (1 μg/μL protein) was mixed with 1 μL of Fluorescent 5 × Master Mix (ProteinSimple) in the presence of fluorescent molecular weight markers and 400 mM dithiothreitol (ProteinSimple). This preparation was denatured at 95 °C for 5 min for Nef, p-Tau217, and flotillin 1 analysis. For ABCA1, the mix was incubated at 37 °C for 15 min. Molecular weight ladder and proteins were separated in capillaries through a separation matrix at 375 V. A ProteinSimple proprietary photoactivated capture chemistry was used to immobilize separated viral proteins on the capillaries. Capillaries with immobilized proteins were blocked with KPL Detector Block (5X) (SeraCare Life Sciences, Gaithersburg, MD, cat. #5920–0004) for 60 min and then incubated with primary antibodies (see the section below) for 60 min. After a wash step, HRP-conjugated goat anti-rabbit (cat. #042–206), donkey anti-goat (cat. #043–522-2), or goat anti-mouse (cat. #042–205) antibodies from ProteinSimple were added for 30 min to capillaries with a corresponding primary antibody. The chemiluminescent revelation was established with peroxide/luminol-S (ProteinSimple). Digital image of chemiluminescence of the capillary was captured with Compass Simple Western software (version 5.1.0, Protein Simple) that calculated automatically heights (chemiluminescence intensity), area, and signal/noise ratio. Results were visualized as electropherograms representing the peak of chemiluminescence intensity and as lane view from the signal of chemiluminescence detected in the capillary. A total protein assay using Total Protein detection module DM-TP01 and Replex Module RP-001 was included in each run to quantitate loading. Samples were analyzed at least 2 times to ensure consistency of the results.

### Primary Antibodies

For Nef detection, the following mouse monoclonal antibodies were used: 3D12 (Abcam, cat. #ab42355) against amino acids ^35^RDLEKHGAITSSNTAA^50^ of HIV-1 HXB2; EH1 (NIH HIV and AIDS Reagent Program, cat. #ARP-3689) against ^194^MARELHPEYYKDC^206^ of HIV-1 B subtype consensus; SN20 (gift from Dr. Bernhard Maier, Indiana University) against the SH3 binding domain of Nef (FPVTPQ); and 6JR (Abcam, cat. # ab42358) mapped to ^195^ARELHPEYYKD^205^ of the HIV-1 B subtype consensus. P-Tau217 was detected using the rabbit polyclonal antibody to Tau phosphorylated on threonine 217 (Thermo Fisher, cat. #44,744); ABCA1—using mouse monoclonal anti-ABCA1 antibody H10 (Abcam, cat. #ab18180); and flotillin 1—using goat anti-flotillin 1 polyclonal antibody (Novus Biologicals, cat. #NB1001043).

### Mass Spectrometry Analysis of Nef

Brain lysates (50 μL each, 1 μg/μL protein) were precipitated with 4 volumes of cold acetone at − 20 °C for 1 h and solubilized in 75 μL 6 M urea dissolved in 0.1 M NH_4_HCO_3_. Protein reduction and alkylation were carried out by incubation in 0.1 M dithiothreitol at 56 °C for 1 h followed by the incubation in 0.15 M iodoacetamide at room temperature for 1 h. Five volumes of ammonium bicarbonate were added to decrease urea concentration to 1 M and proteins were digested in solution with 10 ng/μL Trypsin Gold (Thermo Fisher, mass spectrometry grade, cat. # V5280) at 37 °C overnight. Digested peptides were purified on ZipTips.

Mass spectrometry analysis was performed on an Orbitrap Exploris™ 480 Mass Spectrometer (Thermo Scientific) with the installed Xcalibur software (version 4.4, Thermo Scientific). Samples were separated by nano liquid chromatography (nano-LC) performed on a UltiMate™ 3000 RSLCnano System connected in-line to the mass spectrometer. Purified peptide mixtures were resuspended in 50 μL of 0.1% formic acid (v/v). A 10-μL sample was loaded and washed for 5 min on a C_18_-trap column (0.3 × 5 mm, 5 μm, 100 Å) with a solvent of A:B = 98:2 (A, 0.1% formic acid solution in water; B, 0.1% formic acid solution in 80% acetonitrile) at a constant flow rate of 300 nL/min. Peptides were transferred to a C_18_-packed Aurora series column (25 cm × 75 μm, 1.6 μm, IonOpticks Pty Ltd, Victoria, Australia) and separated with a linear gradient of 5–35 min, 2–25% B; 35–45 min, 25–45% B; 45–50 min, 45–90% B; 50–55 min, 90% B (v/v) at the flow rate of 300 nL/min. The Orbitrap Exploris 480 was operated under data-dependent acquisition mode. The spray voltage and capillary temperature were set to 1.8 kV and 325 °C, respectively. Full-scan mass spectra were acquired at resolution 120,000 with a scan range of 350–1200 m/*z*, RF Lens (%) 40 and normalized AGC target (%) 300. Intensity threshold 5 × 10^3^, charge stage 2–6, and dynamic exclusion 40 s were enabled. Data-dependent MS^2^ scans were carried out with 3-s cycle time, isolation window (*m*/*z*) 1.6, HCD collision energy (%) 35, Orbitrap resolution 15,000, maximum injection time 40 ms, and AGC target (%) 100.

Nano LC-FT/MS raw data were processed by Proteome Discoverer 2.5 (PD 2.5, Thermo Scientific) using the SEQUEST search engine against the Uniprot HIV-1 database (April 29, 2021, 19,780 sequences) at a false discovery cutoff ≤ 1%. A maximum of two missed cleavage sites was allowed. The mass tolerance for the precursor ion was set at 10 ppm and for the fragment on 0.02 Da. Phosphorylation of serine, threonine, and tyrosine were enabled as dynamic modifications, while carbamidomethylation of cysteine was set as fixed modification. Filter settings including minimum peptide length of 6, peptide modification site probability threshold of 75, and peptide-spectrum matches with a delta Cn value of 0.05 were employed.

### Statistics

Statistical analyses including Spearman correlations, Wilcoxon rank-sum tests, Kruskal–Wallis test with DSCF multiple comparisons option, and multivariate linear regression were conducted in SAS v9.4. For multivariate analysis, Box–Cox transformation of variables was performed in SAS v9.4 prior to regression. Simple linear regression analysis and data visualization were conducted in GraphPad PRISM v9. Figure captions describe the statistical test used.

## Results

**Fig. 1:**
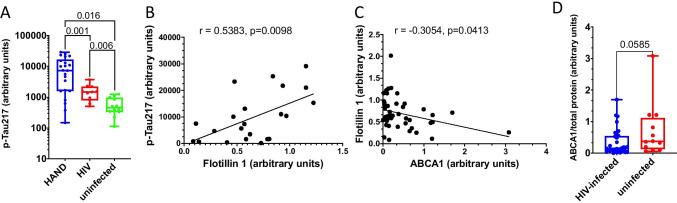
Analysis of pathogenic correlates of HAND. **A** Analysis of p-Tau217 in brain samples from uninfected individuals and HIV-infected individuals with and without HAND diagnosis. Data points for p-Tau217 adjusted to total protein were obtained using ProteinSimple Compass software and are presented as arbitrary units. Group comparisons were made by Kruskal–Wallis test with post hoc Dwass, Steel, Critchlow–Fligner multiple comparison analysis adjustment for FWER (DSCF option in SAS NPAR1WAY procedure). **B** Simple linear regression analysis of p-Tau217 and flotillin 1 in HAND samples (data adjusted to total protein obtained using Compass software). **C** Simple linear regression analysis of ABCA1 and flotillin 1 in all samples (protein abundance adjusted to total protein was obtained using Compass software). *r* and *p* values were calculated by Prism v9 software. *r* and *p* values were calculated by Prism v9 software. **D** Analysis of ABCA1 in HIV-infected (HAND-positive and HAND-negative samples) vs. uninfected samples. Data points representing ABCA1 abundance adjusted to total protein were obtained using Compass and are presented as box and whiskers plot with *p* value calculated by Mann–Whitney nonparametric two-tailed *t* test

The role of amyloid proteins in the pathogenesis of HAND has been previously suggested [25, 32, 42]. Our earlier study of a small number of postmortem brain samples from HAND patients found a trend toward an increased abundance of APP and Tau relative to samples from uninfected individuals [11]. To substantiate this finding, we now analyzed postmortem brain samples from 22 HIV-infected individuals diagnosed with HAND, 11 HIV-infected individuals without HAND diagnosis, and 12 uninfected controls without the diagnosed neurological disease (Table S1) for Tau protein phosphorylated on threonine 217 (p-Tau217). This Tau isoform has been shown to strongly correlate with Aβ deposition [41] and was proposed as a marker for Alzheimer’s disease [21, 27, 36]. Results presented in Fig. 1A demonstrate that the abundance of p-Tau217, measured by quantitative automated capillary Western blot, was significantly increased in samples from HIV-infected individuals with HAND diagnosis, relative to HIV-infected individuals without HAND diagnosis and especially relative to uninfected controls (supporting Western blot evidence is presented in supplementary Fig. S1). A significant difference in the abundance of this peptide was also observed between samples from HIV-infected individuals without HAND diagnosis and uninfected controls (Fig. 1A). Given that p-Tau217 is an early marker of neurodegeneration and Alzheimer’s [21, 27, 36], these results are consistent with the role of phosphorylated Tau in HAND pathogenesis and suggest that increase in this factor may precede HAND diagnosis.

Amyloidogenic proteins and amyloid peptides are associated with lipid rafts [13, 14, 19, 22, 44], and our previous study suggested that increased abundance of lipid rafts caused by Nef-containing extracellular vesicles (exNef) may be the reason for the upregulation of APP and Tau [11]. Unfortunately, immunohistochemical analysis of lipid rafts in fresh-frozen tissue blocks was technically challenging. We evaluated the abundance of lipid rafts in the brain tissue samples by measuring the lipid raft marker flotillin 1 (by quantitative Western blot). Results of these analyses are presented in Fig. S2. There was no statistically significant difference in the abundance of flotillin 1 between brain samples from combined HIV-infected individuals versus uninfected individuals (Fig. S3). However, the abundance of p-Tau217 in HAND-positive brain samples significantly correlated with the abundance of flotillin 1 (Fig. 1B). No such correlation was observed in samples from uninfected brains, or brains from HIV-infected individuals without HAND diagnosis (Fig. S4). This result is consistent with the proposed relationship between lipid rafts and amyloid peptides [11, 14] and makes it likely that only those HIV-infected individuals from our cohort who had an elevated abundance of lipid rafts showed an elevated abundance of pTau and developed HAND.

Results above suggest that the abundance p-Tau217 in HAND brains may be associated with the abundance of flotillin 1 and, by extension, of lipid rafts. Previous studies suggested that downmodulation of ABCA1 in macrophages infected with HIV-1 or treated with exNef regulates the abundance of lipid rafts [2, 9, 11, 34]. Our analysis showed a negative correlation between the quantity of ABCA1 in all samples and the quantity of flotillin 1 (Fig. 1C). Comparison of ABCA1 abundance between all samples from HIV-infected individuals to samples from uninfected controls showed a trend towards reduced ABCA1 in the brains of infected individuals, although the statistical significance was not achieved (Fig. 1D, supporting Western blot evidence shown in Fig. S5).

**Fig. 2:**
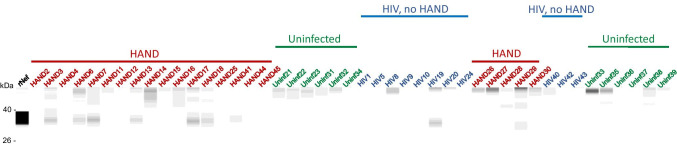
Analysis of Nef in brain samples. Brain lysates were assayed by the Western immunoblot using the 6JR mouse monoclonal anti-Nef antibody from Abcam. Samples were run on the ProteinSimple Jess instrument and analyzed by Compass software. Color-coded lines denote the group assignment of the samples

A known problem with the detection of Nef in biological samples is that Nef proteins from different HIV-1 strains have vastly varying abilities to interact with different antibodies, significantly affecting the accuracy of comparison of the abundance of Nef between infected participants. In addition, the low specificity of most anti-Nef antibodies makes detection of low Nef concentrations in clinical samples challenging. To get around these limitations, we analyzed Nef in the brain samples by Western blot using four different antibodies raised against conservative epitopes in different Nef regions: 3D12 mapped to the N-terminus region of HXB2 Nef, EH1 mapped to the C-terminus region, SN20 mapped to the SH3-binding domain of Nef, and 6JR mapped to the C-terminus region (see “Materials and Methods”). Analysis with 3D12, SN20, and EH1 antibodies was performed with all samples, except HIV 40, HIV 42, HIV 43, HAND 41, HAND 44, and HAND 45, and showed some cross-reactivity with cellular proteins in uninfected samples (Fig. S6). All samples were analyzed using the 6JR antibody, which gave negative results with all uninfected samples (Fig. 2). The results of these experiments are summarized in Table 1.

**Table 1:** Results of Nef analysis in experimental samples

Study ID	SN20	3D12	EH1	6JR	LC–MS/MS	Nef score
HAND 2	−	−	−	−	−	0
HAND 3	+	+	+	+	+	5
HAND 4	−	−	−	−	−	0
HAND 6	+	+	+	+	+	5
HAND 7	+	+	+	+	+	5
HAND 11	+	+	+	−	+	4
HAND 12	−	+	−	−	−	1
HAND 13	+	+	+	+	-	4
HAND 14	−	−	+	−	+	2
HAND 15	−	−	−	−	+	1
HAND 16	+	−	−	−	+	2
HAND 17	+	+	+	+	+	5
HAND 18	−	−	−	+	−	1
HAND 25	−	−	−	−	+	1
HAND 26	+	−	−	−	ND	1
HAND 27	−	−	−	−	ND	0
HAND 28	−	−	+	−	ND	1
HAND 29	+	+	+	+	ND	4
HAND 30	−	−	−	−	ND	0
HAND 41	ND	ND	ND	+	+	2
HAND 44	ND	ND	ND	−	+	1
HAND 45	ND	ND	ND	−	−	0
HIV 1	−	−	−	−	ND	
HIV 5	−	−	−	−	ND	
HIV 8	+	−	−	−	ND	
HIV 9	−	−	−	−	ND	
HIV 10	+	+	−	−	ND	
HIV 19	+	+	+	+	ND	
HIV 20	+	−	−	−	ND	
HIV 24	−	−	−	−	ND	
HIV 40	ND	ND	ND	−	ND	
HIV 42	ND	ND	ND	−	ND	
HIV 43	ND	ND	ND	−	ND	
Uninf 21	+	−	−	−	ND	
Uninf 22	+	−	−	−	ND	
Uninf 23	+	−	−	−	ND	
Uninf 31	+	−	−	−	ND	
Uninf 32	−	−	−	−	ND	
Uninf 33	ND	ND	ND	−	ND	
Uninf 34	−	−	−	−	ND	
Uninf 35	+	−	−	−	ND	
Uninf 36	−	−	−	−	ND	
Uninf 37	+	−	−	−	ND	
Uninf 38	−	−	ND	−	ND	
Uninf 39	+	−	+	−	ND	

*ND*, not done; + , Nef detected; − , Nef undetected

Results of the Western blot analysis were supplemented by tandem liquid chromatography coupled to mass spectrometry (LC–MS/MS analysis) performed on 17 HAND samples for which material remained available. Among the 17 analyzed samples, 11 samples were found to contain Nef-derived peptides (Table S2). Samples HAND3, HAND7 and HAND11 shared the same peptide (accession number A0A0S3QM10), and samples HAND6 and HAND16 shared another peptide (accession number C8C8S7, Table S2). These Nef peptides showed strong MS/MS spectra (Figs. S7A and B), validating the results.

**Fig. 3:**
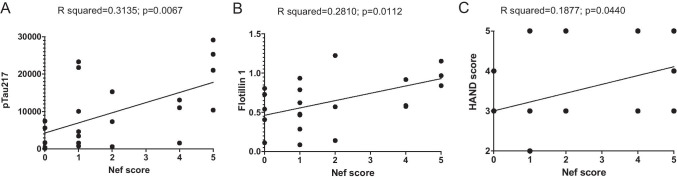
Simple linear regression analysis of samples from HAND-diagnosed individuals. Simple linear regression analysis was performed between Nef score and pTau217 (**A**) and flotillin 1 (**B**), assessed using ProteinSimple Compass software, and presented as arbitrary units. **C** Linear regression between Nef score and HAND clinical scores. *R* squared and *p* values were calculated by Prism v9 software

The sum of positive results in these 5 tests (4 antibodies and MS/MS) allowed us to calculate a Nef score that reflected the likelihood that Nef was present in the sample (Table 1). We performed linear regression analysis with the Nef score and abundance of flotillin 1 and p-Tau217. There was a significant positive correlation between the Nef score and abundance of flotillin 1 (Fig. 3A) and p-Tau217 (Fig. 3B). To determine whether the presence of Nef was associated with the disease status, we correlated the Nef score with the clinical HAND score. Clinical neurological status was provided to us by the NNTC after being assessed using three existing dementia rating scales, American Academy of Neurology (AAN), Memorial Sloan Kettering (MSK), and Frascati; the scales were not universally applied to all individuals and in some cases disagreed (Table S1). Although a good concordance between these scales has been reported, Frascati provided a more graded evaluation, especially for mild cases [17]. We therefore used the Frascati score, where available, to grade the clinical status, and the AAN score in the other cases (Table 2). The HAND clinical score significantly correlated with the Nef score (Fig. 3C), indicating that the presence of Nef was associated with a more severe disease. Indeed, the most severe form of HAND (HIV-associated dementia) was observed only in subjects with the highest Nef score (Fig. 3C and Table 2). Multivariate regression analysis demonstrated that the effect of detectable Nef on all continuous outcome variables (ABCA1, flotillin 1, p-Tau217) considered jointly was significant in all HIV-infected individuals, in both the unadjusted model and the adjusted model controlling for age, HIV duration, and viral load (Table S3). The difference in results between analysis of all samples from HIV-positive individuals and samples from HAND-diagnosed individuals may be due to the smaller sample size in the HAND set.

**Table 2:** HAND severity evaluation in donors of postmortem brain samples^A^

ID	Frascati score	AAN score	HAND score in the study
HAND 2		3	3
HAND 3		5	5
HAND 4		4	4
HAND 6		4	4
HAND 7		5	5
HAND 11		3	3
HAND 12		2	2
HAND 13		5	5
HAND 14		3	3
HAND 15		3	3
HAND 16	3		3
HAND 17	3		3
HAND 18	2		2
HAND 25	3		3
HAND 26		3	3
HAND 27		3	3
HAND 28		3	3
HAND 29		3	3
HAND 30		3	3
HAND 41	5	4	5
HAND 44	5	4	5
HAND 45	3	5	3

^A^HAND score used in the study was based on the Frascati score where available and the AAN score in other cases. Assuming a correlation between the Frascati and AAN scales [17], the HAND scores correspond to the following clinical classification established for the Frascati criteria: 0, neurocognitively normal, no significant impairment on NP testing; 1, asymptomatic neurocognitive impairment; 2, possible mild neurocognitive disorder; 3, probable mild neurocognitive disorder; 4, possible HIV-associated dementia; 5, probable HIV-associated dementia

Taken together, these results show that the presence of Nef is associated with the progression of HAND. Although most findings are associations and do not prove causality, they suggest that Nef, via modification of the lipid rafts, is involved in promoting amyloid formation and disease progression.

## Discussion

The role of amyloid proteins in HAND pathogenesis remains an open question. In this study, we demonstrated a significant increase in the brains of HIV-infected individuals of the abundance of p-Tau217, which is also involved in the pathogenesis of Alzheimer’s disease [7]. This finding is consistent with our previous report where in vitro analysis suggested an increased abundance of amyloid precursor proteins and Tau in exNef-treated neuronal cells [11]. Surprisingly, this was true for brain samples from both HAND-diagnosed and HAND-free HIV-infected individuals. One explanation is that HIV-infected individuals without HAND diagnosis were at an early stage of disease when clinical manifestations could not yet be detected. This explanation is consistent with the demonstrated utility of p-Tau217 as an early marker of Alzheimer’s, which allows detection of the disease at the stage preceding neurodegeneration and clinical symptoms [1].

In these studies, we measured total flotillin 1 to evaluate the abundance of the lipid rafts. Flotillins 1 and 2 are integral membrane proteins that assist in raft assembly [40, 43, 45]. While flotillin 1 can be recruited to lipid rafts under certain stimulatory conditions [18], suggesting that the number of flotillin molecules per lipid raft may vary, the level of flotillin expression, and thus the per cell abundance of flotillin, appears to regulate the abundance of lipid rafts [31]. Therefore, the abundance of total flotillin 1 provides only a crude estimate of the abundance of lipid rafts. This limitation is taken into account in our interpretations.

Tau hyperphosphorylation is a characteristic feature of Alzheimer’s disease, and a number of studies established that phosphorylation of Tau in AD is closely linked to Aβ pathology [4, 28, 41]. Lipid rafts were proposed to be the sites where Tau phosphorylation takes place and where phosphorylated Tau accumulates and interacts with Aβ during the development of Alzheimer’s disease [22]. The observed correlation between the abundances of flotillin 1 and p-Tau217 may reflect this paradigm, suggesting that lipid rafts may be a target for potential therapeutic interventions in HAND, as had been proposed for other neurodegenerative diseases [6]. We recognize, however, that, as discussed above, the abundance of flotillin 1 provides only a crude estimate of the abundance of lipid rafts, which may be the reason for low flotillin 1 in some brain samples from subjects diagnosed with HAND and high flotillin 1 in some samples from HIV-infected individuals without the HAND diagnosis. But the most likely explanation for this variability is that a driving factor in HAND pathogenesis is not the absolute amount, but the change in the quality and quantity of the lipid rafts during disease progression. Our previous studies documented the functional impairment of lipid rafts associated with the effect of Nef [9], which may occur without a major change in the raft abundance. We have previously reported that exNef modify the fatty acid content of the lipid rafts [34], although the functional consequences of these changes have not been investigated. Prospective studies will be needed to evaluate the relationship between structural features of lipid rafts and HAND pathogenesis.

The detection of Nef by immunological methods is challenging due to Nef variability [30]. In addition, the low abundance of Nef and its likely localization to specific brain regions [47] make its detection even more difficult. We used several anti-Nef antibodies and complemented this analysis with LC–MS/MS, allowing us to get a score reflecting the likeliness of Nef presence. Most of the samples where Nef could be detected came from individuals with diagnosed HAND. Moreover, the Nef score significantly correlated with flotillin 1, p-Tau217, and clinical disease score. These results suggest that Nef may drive ABCA1 downmodulation initiating lipid raft modifications and subsequent pathological events exacerbating HAND progression.

A limitation of this study is that a relatively small number of samples is available for analysis. Since samples were not cryopreserved, we could not perform flow cytometry analysis of the lipid rafts, or cell-specific characterizations. Another limitation is the lack of a sensitive quantitative assay for Nef. In future analysis, targeted LC–MS/MS employing single or multiple reaction monitoring might be developed using the information on Nef peptides obtained in our study. Despite these limitations, our study supports the pathogenic role of Tau in HAND pathogenesis and suggests the sequence of events that lead to HAND: Nef EVs downmodulate ABCA1 changing the properties of the lipid rafts, thus increasing the formation of amyloid plaques and Tau phosphorylation and fibrillation. Future studies in animal models will establish the temporality of this ordering and will determine whether therapeutic treatments breaking the pathogenic course described above can prevent the development of HAND.

## Supplementary Information

Below is the link to the electronic supplementary material.

Supplementary file1 (DOCX 15 KB)

Supplementary file2 (PPTX 78387 KB)

Supplementary file3 (PPTX 13554 KB)

Supplementary file4 (PPTX 239 KB)

Supplementary file5 (PPTX 90 KB)

Supplementary file6 (PPTX 8282 KB)

Supplementary file7 (PPTX 35887 KB)

Supplementary file8 (PPTX 263 KB)

Supplementary file9 (DOCX 21 KB)

Supplementary file10 (DOCX 14 KB)

## Data Availability

All the data are presented in the manuscript as the main or supplementary material. The sources of materials are provided.
