# Supplementary material for: Abundance of Nef and p-Tau217 in Brains of Individuals Diagnosed with HIV-Associated Neurocognitive Disorders Correlate with Disease Severance
Source: Mol Neurobiol. Author manuscript; Available in PMC 2022 Feb 23. (PMC8857174; doi:10.1007/s12035-021-02608-2)
Supplement: Supplemental Table 1 [file NIHMS1770521-supplement-Supplemental_Table_1.pdf]

Table S1. Clinical data for the individuals from whom brain samples were obtained.

Original ID[A]	Study ID[B]	Age	Gender	Frascati[C]	AAN	MSK	VL	logVL	HIV duration
1071	HAND 2	39	M		3	1	8,584	3.93	12
4041	HAND 3	34	F		5	2	165,682	5.22	5
4028	HAND 4	46	F		4	2	750,000	5.88	6
6011	HAND 6	49	M		4	2	5,000	3.7	10
6046	HAND 7	50	M		5	2	400	2.6	15
7100118382	HAND 11	37	M		3	2	178	2.25	19
1021	HAND 12	41	F		2	1	400	2.6	5
2066	HAND 13	35	M		5	2	250,000	5.4	13
1159	HAND 14	44	M		3	1	69,931	4.84	13
2074	HAND 15	36	M		3	1	6,952	3.84	3
CE135	HAND 16	39	M	3		1	198,779	5.3	13
CE116	HAND 17	46	F	3			342,386	5.53	13
CC101	HAND 18	44	M	2			39,530	4.6	19
CC202	HAND 25	36	M	3		1	750,000	5.88	UK[D]
7100056883	HAND 26	35	M		3	1	18,985	4.28	6
7100686683	HAND 27	31	M		3	2	483,758	5.68	5
7200077165	HAND 28	45	M		3	1	316,227	5.5	8
7200116866	HAND 29	36	M		3	2	433,715	5.64	5
7200017177	HAND 30	41	M		3	2	501	2.7	16
010123	HAND 41	44	F	5	4	2	129,083	5.11	13
030013	HAND 44	30	M	5	4	2	104,300	5.02	8
030025	HAND 45	46	M	3	5	2	750,000	5.88	3
6052	HIV 1	39	M		0		75,000	4.88	14
4109	HIV 5	32	M		0		400	2.6	4
CE122	HIV 8	39	M	0			229,168	5.36	20
CB194	HIV 9	37	M	0			9,542	3.98	9
7200207472	HIV 10	35	M		0		135,643	5.13	5
CA236	HIV 19	34	M	0			552,000	5.74	8
CC163	HIV 20	45	M	0			16,700	4.22	21
CE119	HIV 24	41	F	0			83,061	4.92	11
010104	HIV 40	50	M	0	3		26,621	4.43	14
010251	HIV 42	40	F	0	4		77	1.89	17
030006	HIV 43	37	M	0			287,947	5.46	4
GW5	uninf 21	55	F						
GW22	uninf 22		F						
GW367	uninf 23	84	M						
HU13350	uninf 31	45	M						
HU13134	uninf 32	53	M						
HU13477	uninf 33	58	M						

HU13345	uninf 34	62	M						
HU13440	uninf 35	68	M						
HU13323	uninf 36	24	F						
HU13344	uninf 37	33	F						
HU13243	uninf 38	53	F						
HU13114	uninf 39	54	F						

[A] Original sample IDs. Samples from HIV-infected individuals with or without HAND diagnosis were provided by NNTC, samples from uninfected individuals came from GWU (GW) or NBB (HU). All clinical data were supplied by the providers. VL – viral load (RNA copies/ml), HIV duration – known length of HIV infection (years).
[B] IDs used in the study. HAND or HIV (no HAND) designation was made based on NNTC grouping.
[C] Frascati scores were assigned by NNTC: 0 - Neurocognitively normal, no significant impairment on NP testing; 1 - Asymptomatic Neurocognitive Impairment; 2 - Possible Mild Neurocognitive Disorder; 3 - Probable Mild Neurocognitive Disorder; 4 - Possible HIV Associated Dementia; 5 - Probable HIV Associated Dementia.
[D] Data not provided by NNTC.
