# Supplementary material for: Abundance of Nef and p-Tau217 in Brains of Individuals Diagnosed with HIV-Associated Neurocognitive Disorders Correlate with Disease Severance
Source: Mol Neurobiol. Author manuscript; Available in PMC 2022 Feb 23. (PMC8857174; doi:10.1007/s12035-021-02608-2)
Supplement: Supplemental Table 2 [file NIHMS1770521-supplement-Supplemental_Table_2.pdf]

Table S2. Accession numbers, Proteome Discoverer 2.5 Scores and top peptide sequences for Nef detected by LC-MS/MS in the HAND samples.

Sample	Accession	PD Score	peptide
HAND2		N/D	
HAND3	A0A0S3QM10	3.11	HC LLHPMNQHGA EDADREVLMW KFDS(p)ALAR
HAND4		N/D	
HAND6	C8C8S7	2.8	LEPVEA EEVEEANKGE NNCLLHPVS(p)L HGIEDPEREV LR
HAND7	A0A0S3QM10	2.79	HC LLHPMNQHGA EDADREVLMW KFDS(p)ALAR
HAND11	A0A0S3QM10	2.70	HC LLHPMNQHGA EDADREVLMW KFDS(p)ALAR
HAND12		N/D	
HAND13		N/D	
HAND14	A0A0S3QM10	2.42	HC LLHPMNQHGA EDADREVLMW KFDS(p)ALAR
HAND15	J3R288	2.88	KAEPGEPGE PAADGVGAVS (p) RDLEK
HAND16	C8C8S7	2.98	LEPVEA EEVEEANKGE NNCLLHPVS(p)L HGIEDPEREV LR
HAND17	A0A088LIZ2	2.72	QAEPAREPA AVGVGAAS(p)QD LDK
HAND18		N/D	
HAND25	C8C9H1	2.52	MR QAEPEPAAAG VGAVS(p)RDLEK
HAND41	E0WWI5	2.92	R NEPAVEPAAV GVGAVS(p)RDLA ER
HAND44	A0A248XAX7	2.23	RE ERWHAVR
HAND45		N/D	

ND – not detected.
