# Supplementary material for: Abundance of Nef and p-Tau217 in Brains of Individuals Diagnosed with HIV-Associated Neurocognitive Disorders Correlate with Disease Severance
Source: Mol Neurobiol. Author manuscript; Available in PMC 2022 Feb 23. (PMC8857174; doi:10.1007/s12035-021-02608-2)
Supplement: Supplemental Fig 2 [file NIHMS1770521-supplement-Supplemental_Fig_2.pdf]

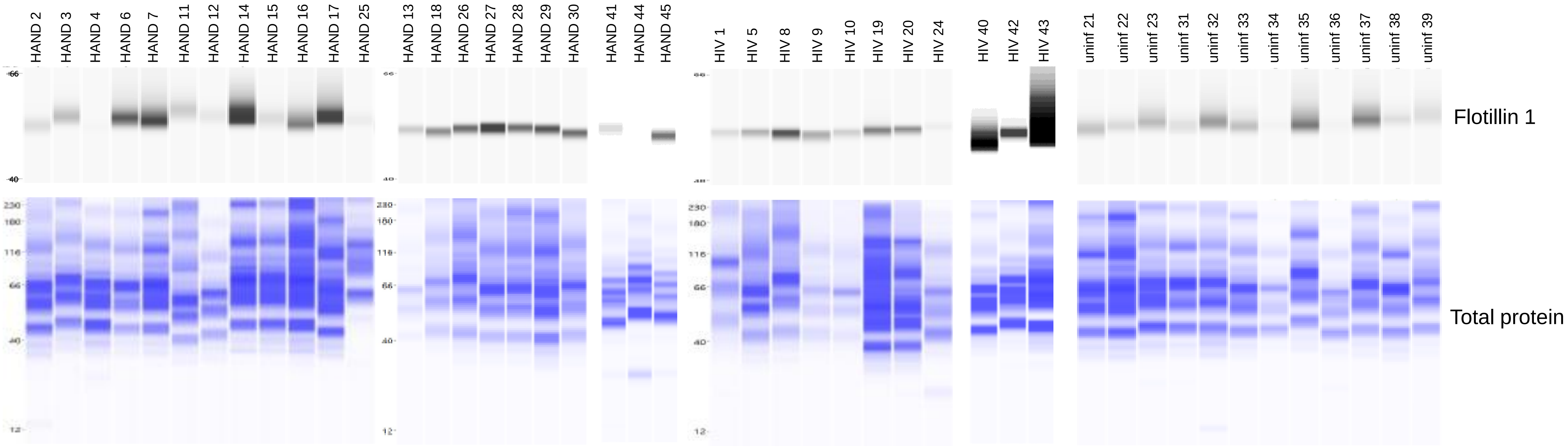

Figure S2. Western blots for Flotillin 1 and total proteins. Molecular weight markers are shown on the left. Position of the flotillin 1 band slightly varies between the capillaries.
