# Supplementary material for: Abundance of Nef and p-Tau217 in Brains of Individuals Diagnosed with HIV-Associated Neurocognitive Disorders Correlate with Disease Severance
Source: Mol Neurobiol. Author manuscript; Available in PMC 2022 Feb 23. (PMC8857174; doi:10.1007/s12035-021-02608-2)
Supplement: Supplemental Fig 4 [file NIHMS1770521-supplement-Supplemental_Fig_4.pdf]

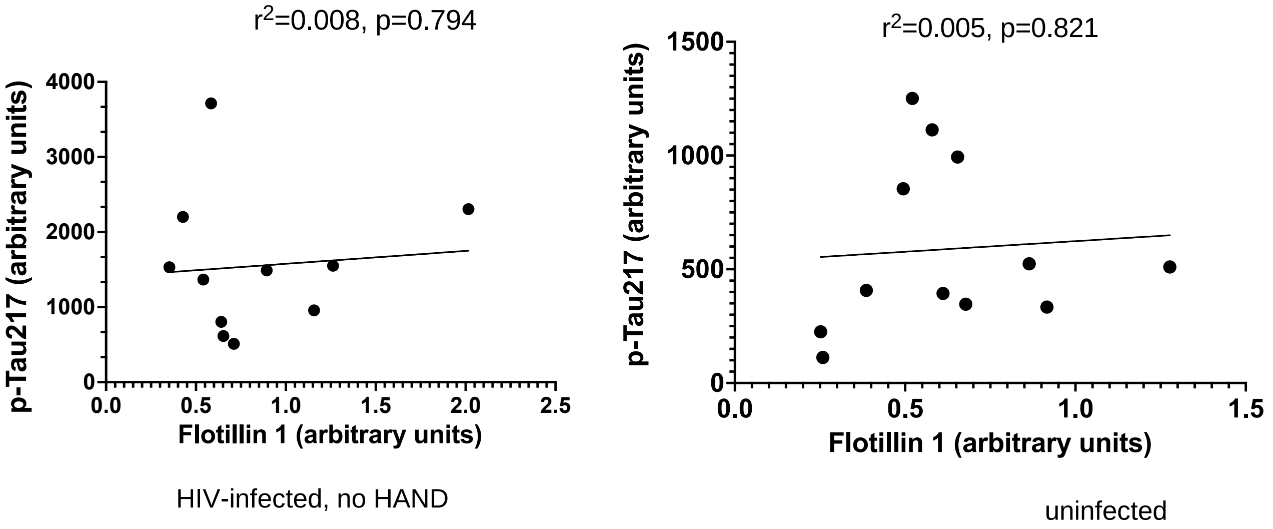

Figure S4. Simple linear regression analysis of p-Tau217 and flotillin 1. Analysis was performed using the GraphPad Prism v.9 software.
