# Supplementary material for: Abundance of Nef and p-Tau217 in Brains of Individuals Diagnosed with HIV-Associated Neurocognitive Disorders Correlate with Disease Severance
Source: Mol Neurobiol. Author manuscript; Available in PMC 2022 Feb 23. (PMC8857174; doi:10.1007/s12035-021-02608-2)
Supplement: Supplemental Fig 3 [file NIHMS1770521-supplement-Supplemental_Fig_3.pdf]

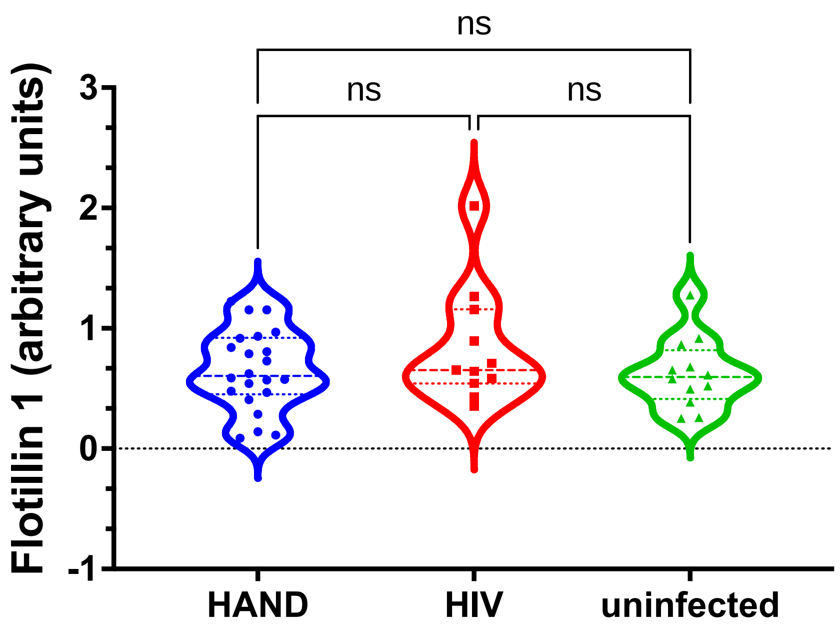

Figure S3. Comparison of Flotillin 1 abundance between brain samples from HIV-infected individuals with (HAND) and without (HIV) HAND diagnosis and uninfected individuals. Data points for Flotillin 1 adjusted to total protein levels were obtained using ProteinSimple Compass software and are presented as arbitrary units. Results are presented as violin plots, p values were calculated using Kruskal-Wallis test.
