# Supplementary material for: Abundance of Nef and p-Tau217 in Brains of Individuals Diagnosed with HIV-Associated Neurocognitive Disorders Correlate with Disease Severance
Source: Mol Neurobiol. Author manuscript; Available in PMC 2022 Feb 23. (PMC8857174; doi:10.1007/s12035-021-02608-2)
Supplement: Supplemental Fig 5 [file NIHMS1770521-supplement-Supplemental_Fig_5.pdf]

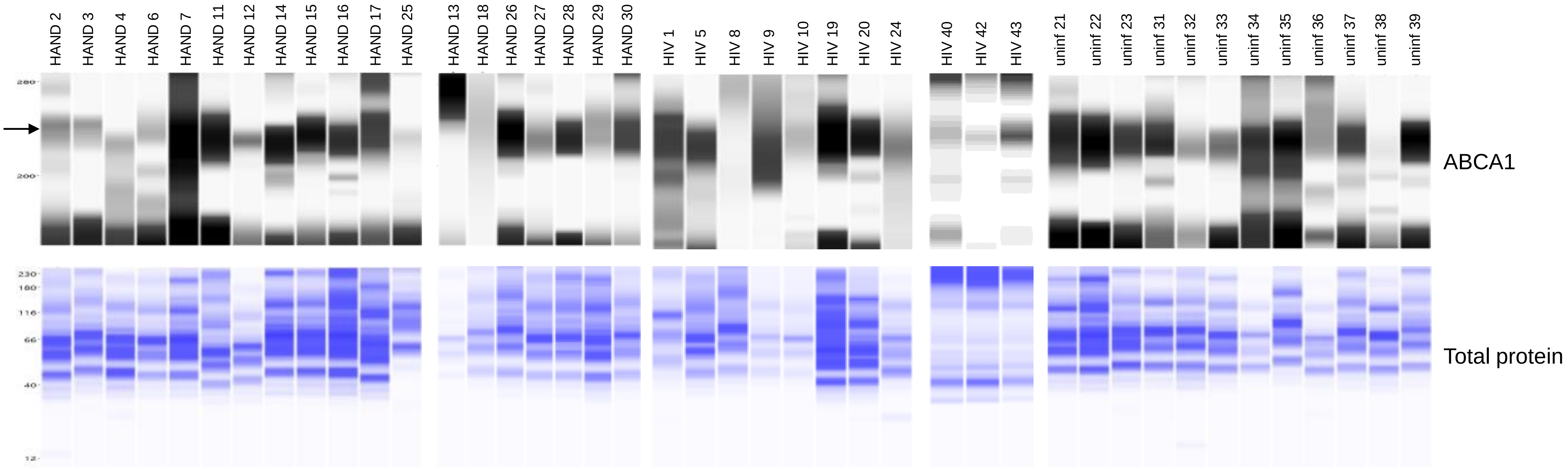

Figure S5. Western blots for ABCA1 and total proteins. Molecular weight markers are shown on the left. The arrow shows position of the ABCA1 band, which runs as a smear due to glycosylation. Migration of the band slightly differs between the capillaries.
