# Supplementary material for: Abundance of Nef and p-Tau217 in Brains of Individuals Diagnosed with HIV-Associated Neurocognitive Disorders Correlate with Disease Severance
Source: Mol Neurobiol. Author manuscript; Available in PMC 2022 Feb 23. (PMC8857174; doi:10.1007/s12035-021-02608-2)

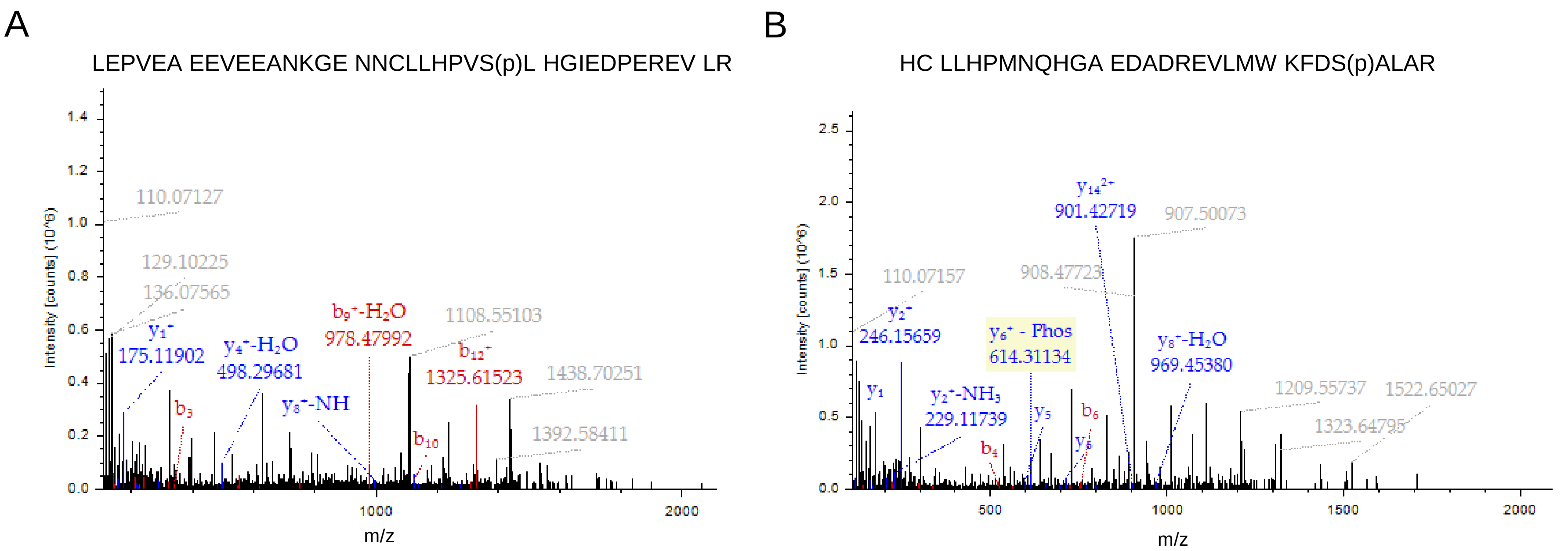

Figure S7. Nef detection in HAND samples by LC-MS/MS. A and B - MS/MS spectra of the Nef peptides in the HAND samples 6, 16 and 3, 7, 11, respectively. The colored peaks indicate matched MS/MS fragments. Blue and red colors indicate y and b ions, respectively. The spectrum gives positive identification of peptides LEPVEA EEVEEANKGE NNCLLHPVS(p)L HGIEDPEREV LR (panel A) and HC LLHPMNQHGA EDADREVLMW KFDS(p)ALAR (panel B) with the indicated phosphorylation sites. The peptides matched to the Nef proteins with accession numbers C8C8S7 and A0A0S3QM10, respectively.

Fig. S6

A

HC LLHPMNQHGA EDADREVLMW KFDS(p)ALAR

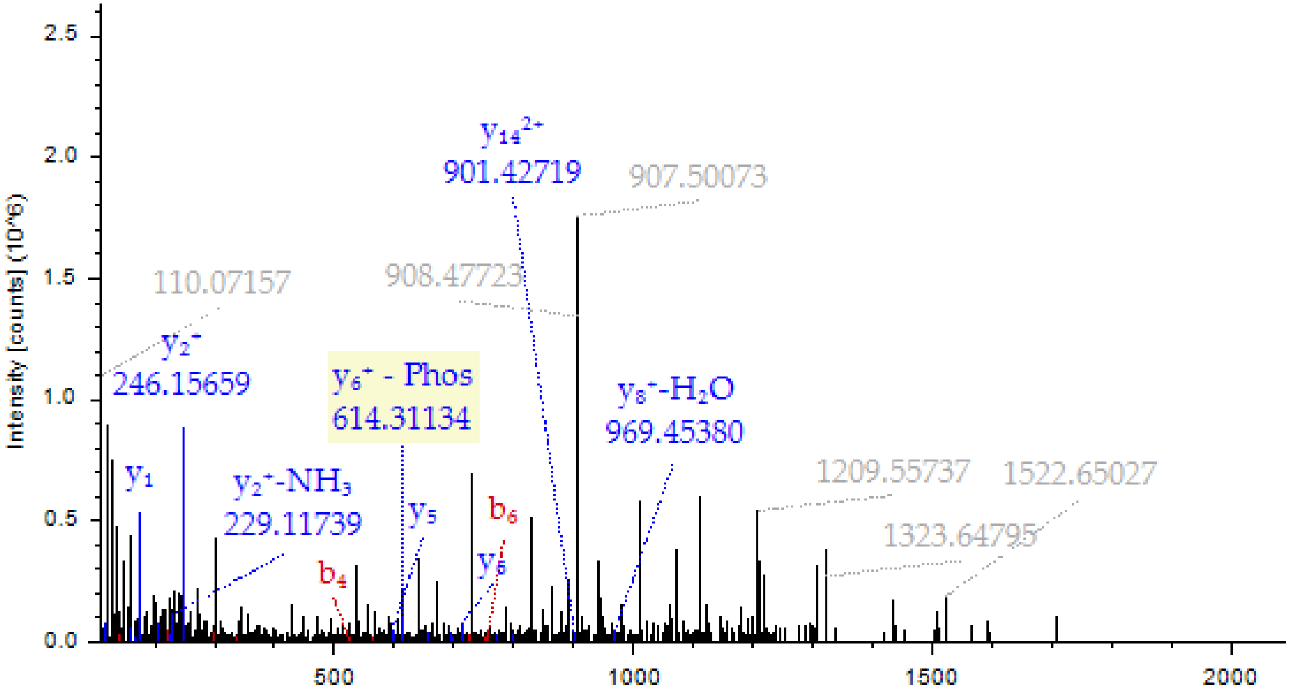

B

LEPVEA EEVEEANKGE NNCLLHPVS(p)L HGIEDPEREV LR

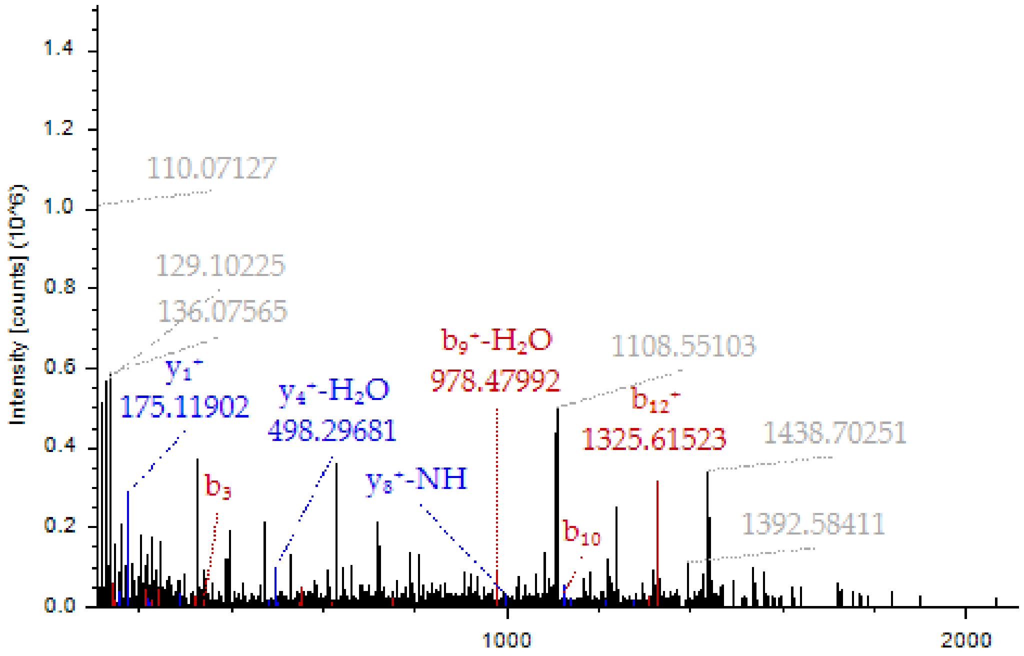

Supplement: Supplemental Fig 7 [file NIHMS1770521-supplement-Supplemental_Fig_7.pdf]
