# Supplementary material for: Abundance of Nef and p-Tau217 in Brains of Individuals Diagnosed with HIV-Associated Neurocognitive Disorders Correlate with Disease Severance
Source: Mol Neurobiol. Author manuscript; Available in PMC 2022 Feb 23. (PMC8857174; doi:10.1007/s12035-021-02608-2)
Supplement: Supplemental Fig 6 [file NIHMS1770521-supplement-Supplemental_Fig_6.pdf]

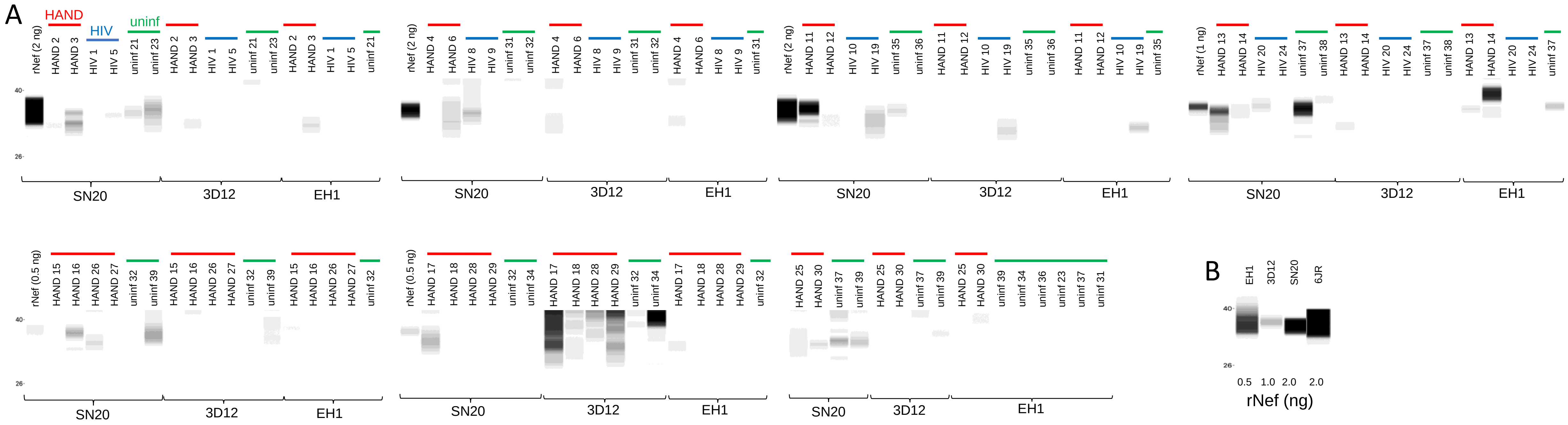

Figure S6. Analysis of Nef in brain samples. Brain lysates were analyzed on ProteinSimple Jess instrument using the Compass software. A – Sample IDs are shown on top of the gels, and antibodies used for detection – below the gels. B – Detection of recombinant Nef from HIV-1_{SF2} by antibodies used in this study. Color coded lines show the sample group.
