# Supplementary material for: Abundance of Nef and p-Tau217 in Brains of Individuals Diagnosed with HIV-Associated Neurocognitive Disorders Correlate with Disease Severance
Source: Mol Neurobiol. Author manuscript; Available in PMC 2022 Feb 23. (PMC8857174; doi:10.1007/s12035-021-02608-2)
Supplement: Supplemental Table 3 [file NIHMS1770521-supplement-Supplemental__Table_3.pdf]

Table S3. Multivariate linear regression results for Nef (undetectable vs. detectable) predicting outcomes										
**Outcome Variable[A]**	**Estimate**	**95% LCL**	**95% UCL**	**p-Value**	**MANOVA p-Value[B]**	**Adjusted Estimate[C]**	**Adjusted 95% LCL**	**Adjusted 95% UCL**	**Adjusted p-Value**	**Adjusted MANOVA p-Value**
Participants with HAND (n=22)										
ABCA1[D]	-0.22	-0.49	0.05	0.105	-	-0.15	-0.46	0.17	0.331	-
Flotillin-1	0.26	-0.02	0.54	0.070	-	0.19	-0.16	0.53	0.271	-
p-Tau217[E]	0.48	-0.08	1.04	0.089	-	0.33	-0.24	1.14	0.183	-
Overall model	-	-	-	-	0.051	-	-	-	-	0.304
All participants with HIV (n=33)										
ABCA1[D]	-0.14	-0.39	0.10	0.244	-	-0.10	-0.37	0.16	0.423	-
Flotillin-1	0.10	-0.21	0.40	0.511	-	0.08	-0.25	0.41	0.634	-
p-Tau217[E]	**0.62**	**0.20**	**1.04**	**0.005**	-	**0.65**	**0.20**	**1.09**	**0.006**	-
Overall model	-	-	-	-	**0.014**	-	-	-	-	**0.030**
[A]Modeling effect of the predictor Nef = detectable (undetectable as the reference category)										
[B]MANOVA p-value by Pillai's trace exact F test for the overall effect of Nef										
[C]Controlling for age, HIV duration (in years), and log_{10} viral load										
[D]Modeling square root of ABCA1 values										
[E]Modeling log_{10} p-Tau217 values										
Note: significant effects at the alpha=0.05 level are bolded										
LCL - lower confidence limit; UCL - upper confidence limit; MANOVA - multivariate analysis of variance										
