# Supplementary material for: Abundance of Nef and p-Tau217 in Brains of Individuals Diagnosed with HIV-Associated Neurocognitive Disorders Correlate with Disease Severance
Source: Mol Neurobiol. 2021 Nov 29;59(2):1088–97. doi: 10.1007/s12035-021-02608-2 (PMC8857174; doi:10.1007/s12035-021-02608-2)
Supplement: Supplementary file 1 [file 12035_2021_2608_MOESM10_ESM.docx]

**Table S2. Accession numbers, Proteome Discoverer 2.5 Scores and top peptide sequences for Nef detected by LC-MS/MS in the HAND samples.** 

| Sample | Accession | PD Score | peptide |
| --- | --- | --- | --- |
| HAND2 |  | N/D |  |
| HAND3 | A0A0S3QM10 | 3.11 | HC LLHPMNQHGA EDADREVLMW KFDS(p)ALAR |
| HAND4 |  | N/D |  |
| HAND6 | C8C8S7 | 2.8 | LEPVEA EEVEEANKGE NNCLLHPVS(p)L HGIEDPEREV LR |
| HAND7 | A0A0S3QM10 | 2.79 | HC LLHPMNQHGA EDADREVLMW KFDS(p)ALAR |
| HAND11 | A0A0S3QM10 | 2.70 | HC LLHPMNQHGA EDADREVLMW KFDS(p)ALAR |
| HAND12 |  | N/D |  |
| HAND13 |  | N/D |  |
| HAND14 | A0A0S3QM10 | 2.42 | HC LLHPMNQHGA EDADREVLMW KFDS(p)ALAR |
| HAND15 | J3R288 | 2.88 | KAEPGEPGE PAADGVGAVS (p) RDLEK |
| HAND16 | C8C8S7 | 2.98 | LEPVEA EEVEEANKGE NNCLLHPVS(p)L HGIEDPEREV LR |
| HAND17 | A0A088LIZ2 | 2.72 | QAEPAREPA AVGVGAAS(p)QD LDK |
| HAND18 |  | N/D |  |
| HAND25 | C8C9H1 | 2.52 | MR QAEPEPAAAG VGAVS(p)RDLEK |
| HAND41 | E0WWI5 | 2.92 | R NEPAVEPAAV GVGAVS(p)RDLA ER |
| HAND44 | A0A248XAX7 | 2.23 | RE ERWHAVR |
| HAND45 |  | N/D |  |

ND – not detected.
