# Supplementary material for: Abundance of Nef and p-Tau217 in Brains of Individuals Diagnosed with HIV-Associated Neurocognitive Disorders Correlate with Disease Severance
Source: Mol Neurobiol. 2021 Nov 29;59(2):1088–97. doi: 10.1007/s12035-021-02608-2 (PMC8857174; doi:10.1007/s12035-021-02608-2)
Supplement: Supplementary file 2 [file 12035_2021_2608_MOESM1_ESM.docx]

| **Table S****3****. Multivariate linear regression results for Nef (undetectable vs. detectable) predicting outcomes** |  |  |  |  |  |  |  |  |  |  |
| --- | --- | --- | --- | --- | --- | --- | --- | --- | --- | --- |
| **Outcome Variable**^**A**^ | **Estimate** | **95% LCL** | **95% UCL** | **p-Value** | **MANOVA p-Value**^**B**^ | **Adjusted Estimate**^**C**^ | **Adjusted 95% LCL** | **Adjusted 95% UCL** | **Adjusted p-Value** | **Adjusted MANOVA p-Value** |
| *Participants with HAND (n=22)* |  |  |  |  |  |  |  |  |  |  |
| ABCA1^D^ | -0.22 | -0.49 | 0.05 | 0.105 | - | -0.15 | -0.46 | 0.17 | 0.331 | - |
| Flotillin-1 | 0.26 | -0.02 | 0.54 | 0.070 | - | 0.19 | -0.16 | 0.53 | 0.271 | - |
| p-Tau217^E^ | 0.48 | -0.08 | 1.04 | 0.089 | - | 0.33 | -0.24 | 1.14 | 0.183 | - |
| Overall model | - | - | - | - | 0.051 | - | - | - | - | 0.304 |
| *All participants with HIV (n=33)* |  |  |  |  |  |  |  |  |  |  |
| ABCA1^D^ | -0.14 | -0.39 | 0.10 | 0.244 | - | -0.10 | -0.37 | 0.16 | 0.423 | - |
| Flotillin-1 | 0.10 | -0.21 | 0.40 | 0.511 | - | 0.08 | -0.25 | 0.41 | 0.634 | - |
| p-Tau217^E^ | **0.62** | **0.20** | **1.04** | **0.005** | - | **0.65** | **0.20** | **1.09** | **0.006** | - |
| Overall model | - | - | - | - | **0.014** | - | - | - | - | **0.030** |
| ^A^Modeling effect of the predictor Nef = detectable (undetectable as the reference category) |  |  |  |  |  |  |  |  |  |  |
| ^B^MANOVA p-value by Pillai's trace exact F test for the overall effect of Nef |  |  |  |  |  |  |  |  |  |  |
| ^C^Controlling for age, HIV duration (in years), and log_10_ viral load |  |  |  |  |  |  |  |  |  |  |
| ^D^Modeling square root of ABCA1 values |  |  |  |  |  |  |  |  |  |  |
| ^E^Modeling log_10_ p-Tau217 values |  |  |  |  |  |  |  |  |  |  |
| Note: significant effects at the alpha=0.05 level are bolded |  |  |  |  |  |  |  |  |  |  |
| LCL - lower confidence limit; UCL - upper confidence limit; MANOVA - multivariate analysis of variance |  |  |  |  |  |  |  |  |  |  |
